# Prevalence of Stroke in Neonates Who Admitted With Seizures in Neonatal Intensive Care Unit

**Published:** 2015

**Authors:** Roya FARHADI, Abdolrasool ALAEE, Zahra ALIPOUR, Ali ABBASKHANIAN, Maryam NAKHSHAB, Hojjat DERAKHSHANFAR

**Affiliations:** 1 Pediatrics Department, Mazandaran University of Medical Sciences, Sari, Iran; 2 Radiology Department, Mazandaran University of Medical Sciences, Sari, Iran; 3 Faculty of Medicine, Mazandaran University of Medical Sciences, Sari, Iran; 4 Pediatric Neurology Department, Mazandaran University of Medical Sciences, Sari, Iran; 5 Pediatrician, Emergency Medicine Department, Shahid Beheshti University of Medical Sciences, Tehran, Iran

**Keywords:** Stroke, Neonate, Seizure, Transcranial Doppler Sonography

## Abstract

**Objective:**

Prevalence of neonatal stroke has been reported 1/2300-1/4000 live births and accounts for 12-20% of the cases of neonatal seizures. Although stroke has been introduced as the second cause of the neonatal seizures in literatures, it may remain unclear in diagnostic evaluations of seizure in neonates. This study was performed to assess the prevalence of stroke in neonates with seizure.

**Materials & Methods:**

In this cross-sectional study, all neonates ≥ 28 weeks of gestation with a diagnosis of seizures admitted to the NICU of Boo-Ali Sina Hospital in Sari, north of Iran, were enrolled. Brain CT scan and a Transcranial Doppler ultrasonography were performed for the all cases. In cases that stroke were reported in one or two above modalities, an MRI was also performed and prevalence of stroke was reported. Putative risk factors of stroke were analyzed with univariate and multivariate statistical methods.

**Results:**

From 174 newborn infants, 75.3% of neonates were male. Prevalence of stroke was 8%, 2.3% and 3.4% in Doppler ultrasonography, CT scan and MRI reports respectively. Umbilical venous catheterization was the risk factor of stroke in the univariate and multivariate analysis (P= 0.001; OR, 10.39; 95% CI, 2.72- 39.77). The most common form of seizure was focal clonic seizures (78.6%) in neonates with stroke.

**Conclusion:**

Investigation of stroke as an etiology of neonatal seizures is essential because seizure may be the only symptom of neonatal cerebral infarction. Doppler ultrasonography can be a valuable diagnostic tool at first in critically ill neonates or in situations that MRI is not available primarily. Further studies with notice to outcome assessment of these infants recommended.

## Introduction

Neonatal seizure is one of the most important causes of neurological impairment in newborn infants (1). Identification of the causes of neonatal seizure is essential because the clinical outcomes and management varies in different conditions and appropriate treatment will be required depending on the underlying etiologies (2). Neonatal stroke is a cerebrovascular event occurring in neonatal period at birth to 28 days of postnatal and can be diagnosed with radiological evidence of focal vascular infarction (3). Stroke has been reported in 12-20% of cases of neonatal seizures and occurs in 1/4000 live births. It is an increasingly recognized condition and has been introduced as a second cause of neonatal seizures (3- 6). Seizure is the most common clinical presentation of neonatal stroke and may be the only manifestation in 70-91% of neonates with stroke (7). Although seizure during the first days of life is the usual manifestation of neonatal stroke, it is frequently remained underdiagnosed because most of the affected newborns are asymptomatic and hemi paresis is an uncommon presentation of stroke during neonatal period (7-9). In addition, it can be misdiagnosed with hypoxic ischemic encephalopathy because of similarity of clinical presentations (7). Although the etiologies of neonatal cerebral infarction are complex and are not fully recognized, major risk factors included congenital heart diseases, sepsis, birth trauma, embolism, prothrombotic disease and prenatal risk factors such as chorioamnionitis, prolonged rupture of membranes. Recently, an association between neonatal cerebral infarction and portal vein thrombosis due to umbilical venous catheterization has been reported (3, 9, 10). The middle cerebral artery (MCA), predominantly left MCA is the common affected artery in neonatal stroke (11). Although MRI particularly diffuse weight MRI is considered the imaging method of choice for diagnosis of neonatal stroke, Doppler sonography is also a worthy modality for measuring of the cerebral blood flow and demonstrating the decreases in cerebral blood flow (12, 13). In fact, Doppler sonography plays an important role in initial diagnosis of neonatal stroke as an available screening tool and computed tomography or magnetic resonance imaging is used for definite diagnosis (14). Brain CT scan has a low sensitivity for detecting the cerebral infarction and exposes the neonate to radiation, so it is not the modality of choice for diagnosis of neonatal cerebral infarction (2). However, volumetric techniques are increasingly used to investigate neonatal cerebral injuries. Transcranial ultrasound has been introduced as a non-invasive modality for imaging of blood flow in the major intracranial arteries and is a useful method for detection and following up of the degree of stenosis of great cerebral arteries (15-17). On the other hand, neuroimaging is not easily straight forward, especially in neonates who are unstable and safely transport of them for imaging with CT or preferably, MRI can be difficult. In these conditions, Doppler intracranial sonography can be a helpful non-invasive accessible modality for the evaluation of cerebral vascular infarctions. CT and MRI without diffusion may be normal in early phase of neonatal arterial ischemia (7). Early diagnosis of stroke increases opportunity for specific management strategy (7). Gender and racial differences has been reported for pediatric stroke, but such documents are not available for newborns (9). To the best of our knowledge, no study has reported the prevalence of neonatal stroke as an etiology of seizure among neonatal population in Iran. We decided to investigate the prevalence of stroke in newborn infants who admitted in our NICU with diagnosis of seizure.

**Table 1:** Characteristics of neonates with stroke

Patient	Type of seizure	Apgar score	Type of delivery	Birth weight	Age	Gestational age	sex	*CHD	Mother’s Diabetes mellitus	Umbilical catheterization	Stroke in CT scan	Stroke in MRI
1	Clonic	8	C/S	4000	28	term	Male					+
2	Clonic	8	NVD	4000	28	term	Male				+	
3	Clonic	10	NVD	3500	3	term	Male					
4	Clonic	10	C/S	3690	3	term	Male	+	+	+		
5	Myoclonic	8	C/S	1800	1	preterm	Male				+	
6	Clonic	8	NVD	4000	24	term	Male					+
7	Clonic	10	C/S	3800	1	term	Female	+		+		+
8	Clonic	10	NVD	3500	16	preterm	Male			+		+
9	Clonic	10	C/S	3200	16	preterm	Male					
10	Clonic	9	C/S	3300	8	preterm	Female			+	+	
11	Subtle	10	C/S	3100	14	term	Female					+
12	Subtle	8	C/S	3590	1	preterm	Male		+	+	+	
13	Clonic	8	C/S	1800	1	preterm	Male			+		
14	Clonic	8	NVD	4000	24	term	Male		+	+		+

* CHD= Congenital Heart Diseases

**Table 2:** Related risk factors for stroke in univariate logistic regression analysis

**Risk Factors**	**N(percentage)**		**OddsRatio**	**95% CI**	***P*** ** value**
Gestational age	Stroke (14)	No stroke(160)	*1.20	(0.39-3.62)	0.74
Term	8(57.1)	81(50.6)			
Preterm	6(42.9)	73(45.6)			
Post-term	0(0)	6(3.8)			
Congenital heart diseases			2.25	(0.44-11.37)	0.32
Yes	2(14.3)	11(6.9)			
No	12(85.7)	149(93.1)			
Mother’s diabetes mellitus			3.36	(0.82-13.71)	0.09
Yes	3(21.4)	12(7.5)			
No	11(78.6)	148(92.5)			
Umbilical Catheterization			8.41	(2.63-26.88)	<0.001
Yes	7(50)	17(10.6)			
No	7(50)	143(89.4)			

* Term versus preterm and post-term

**Table 3:** Multivariate logistic regression analysis of risk factors for stroke

**Risk Factors**	**Odds Ratio**	**95% CI**	***P*** ** value**
Gestational age	*2.14	(0.56-8.10)	0.25
Congenital heart disease	1.38	(0.20-9.29)	0.73
Mother’s diabetes melitus	2.15	(0.43-10.56)	0.34
Umbilical catheterization	10.39	(2.72-39.77)	0.001

* Term versus preterm and post-term

## Materials & Methods

This prospective cross sectional study was carried out from February 2012 to December 2013. All neonates≥ 28 weeks of gestation who had a first diagnosis of neonatal seizure or a clinical evident seizure (from birth until 28 days of life) during admission at the NICU of Boo- Ali Sina (level III teaching hospital, Sari, north of Iran) comprised the study population. The study was approved by Ethical Committee of Mazandaran University of Medical Sciences, Iran. A written consent form was obtained from parents before participation of the newborns in the study. Newborn infants with diagnosis of seizure due to hypoglycemia or hypocalcemia with a good response to treatment, and neonates who had not a stable condition for conducting a neuroimaging during the neonatal period were excluded. For all participants, diagnostic investigations for seizure such as clinical and neurological assessment, lumbar puncture, check of serum electrolytes and sepsis workup were performed. Once the patient’s clinical condition stabilized a brain Computed Tomography was carried out as a part of routine evaluation process for seizure. While the neonate was stable and seizure free at least for 48 h, a Doppler transcranial sonography performed. Transcranial Doppler sonography was carried out via fontanel by radiologist of the team who was expert in brain Doppler sonography of neonates and children. The sonography machine used was Siemens G50 with special probe and 12 MHz frequencies. Perfusion index (PI) and resistance index (RI) in middle cerebral artery were measured and abnormal flow or radiological evidence of flow arterial infarction was reported as stroke (3). Then in neonates who had a sonographic scan compatible with diagnosis of stroke, whenever the baby was stable enough to be moved a Diffuse-Weighted (DWI) MRI was carried out for confirmation of the result of transcranial Doppler sonography at least three days after the last episode of seizure. The MRI and CT scan were interpreted by a radiologist, reviewed by a pediatric neurologist, and reevaluated for diagnosis of neonatal stroke. An echocardiography performed for all neonates with a diagnosis of stroke in sonography. Patient’s information including age, sex, birth weight, gestational age, type of delivery, clinical features of seizure (clonic, tonic, subtle or myoclonic), congenital heart disease according to the results of echocardiography, birth trauma, umbilical venous catheterization, polycythemia, coagulation disorders, results of EEG in addition to mother’s prenatal history such as diabetes mellitus, prolonged preterm rupture of membrane (PPROM) and chorioamnionitis were documented.

**Statistical analysis**

Data were analyzed by SPSS version 20 (Chicago, IL, USA) using descriptive analytic statistical tests and reported as mean ± SD and prevalence. For analysis of dichotomous variables, the chi-squared test and fisher exact test were used. P value ≥ 0.05 was considered significant. Logistic regression analysis was used for determining correlation between the related risk factors and the outcome (stroke/ no stroke).

## Results

Overall, 174 neonates born at ≥ 28 weeks gestation who had inclusion criteria of our study were investigated for neonatal seizures. Of these, 89 (51.1%) were term, 79 (45.4%) were preterm, 6 (3.5%) were post-term and 131 (75.3%) were male. Mean birth weight in neonates was 2724.31±828.28 grams and mean of admission age was 10.66± 11.89 days. Cerebral infarction was reported in 14 (8%) of total number of infants with seizures according to Transcranial Doppler sonography. Computed tomography in 4 (2.3%) and MRI in 6 (3.4%) of these cases showed radiographic findings of stroke. The characteristics of 14 individual neonates with stroke are shown in Table 1. Clinical features of seizure were focal clonic in 11(78.6%), subtle in 2 (14.3%) and myoclonic in 1(7.1%) of neonates with stroke. None of these patients showed tonic seizures. In assessment of neonates with stroke, none of them had polycythemia, birth trauma and history of prolonged rupture of membrane or chorioamnionitis in mother. In addition, EEG was abnormal in all cases of stroke. Impairment in initial coagulation tests such as Prothrombin time (PT) or Partial thromboplastin time (PTT) were only observed in two patients of stroke group and these tests were normal in all neonates without stroke. In univariate analysis, umbilical venous catheterization was the only important risk factor for the diagnosis of stroke (P< 0.0001; OR, 8.41; 95%CI, 2.63-26.88). Other investigated predictors such as gestational age, underline congenital heart diseases and history of diabetes mellitus in mother were not significantly different between stroke or non-stroke group (Table2). Using multivariate logistic regression analysis significant related risk factor for stroke included umbilical venous catheterization (P= 0.001; OR, 10.39; 95% CI, 2.72-39.77) (Table 3).

## Discussion

Based on our knowledge, this is the first study that reported the incidence of stroke in neonates with seizures in Iran. The results of our study revealed that 8% of neonates with seizures had ischemia of middle cerebral artery in transcranial Doppler ultrasonography. In previous studies, the incidence of stroke was reported about 12-17.5% in neonates with seizures (6, 9). Gender and racial differences have been identified for stroke in pediatrics, but such information is not available about stroke in neonates (6, 9). The Canadian stroke registry, has reported 12-14% of patients with seizures in the neonatal period were found to have stroke (18). Twelve percent of neonatal seizures were due to stroke (5). This prevalence in Lynch et al. study was reported in up to 20% of neonatal seizures (6). However, this fact is clear that seizure is the most common presentation of neonatal stroke (18). Perlman et al. assessed the clinical characteristics of 8 term infants with neonatal stroke diagnosed by utilizing duplex Doppler according to decreases of blood flow in cerebral arteries and found that 6 of these neonates had seizures and 2 of them presented with apnea and hypotonia (13). In our study, the prevalence of stroke according to CT scanning and Diffusion –Weighted MRI results was 2.3% and 3.4% respectively. Doppler ultrasonography has an important role in initial diagnosis of neonatal cerebral infarction and definite diagnosis is confirmed by CT scan or MRI (14). In other words, Doppler ultrasonography is not diagnostic modality of choice but can be a guiding tool for further diagnostic study.Volumetric techniques are increasingly used to investigate the neonatal brain injuries (15). The middle cerebral artery (MCA) is the most common place of involvement in neonatal stroke and is relatively easy to study, so Doppler ultrasonography has a sensitivity and specificity of 90-99% for detecting an occlusion or stenosis and is correlates well with cerebral angiography (16, 17). Perlman et al. similar to our study used Doppler ultrasonography for measurement of cerebral blood flow velocity then the diagnosis of stroke confirmed by neuroimaging such as MRI (13). Eleven of 14 cases of neonatal stroke detected by MRI were also detected by ultrasonography (19). Shahnaz et al. in a review about neonatal stroke introduced all CT scan, MRI and ultrasonography as diagnostic modalities in detection of cerebral vessels infarction. Their results revealed that although CT scan had better sensitivity than ultrasonography, it might not be able to identify the abnormalities in the early phase of an acute ischemia in neonates. They also believed that Doppler ultrasonography was a most widely used form modality for screening (18). Focal seizures are the most common form of seizures, which has been seen, in neonatal stroke and typically are contralateral to the infarct (20). Similarly, in our study focal clonic seizures were the most type of seizures observed in 78.6% of cases of neonatal stroke. Gelfand et al. reported a case of neonatal focal clonic seizure that showed acute infarct of the angular branch of the right MCA and they confirmed that seizures due to neonatal stroke were typically focal motor seizures (2). Similarly, Rafay et al. in a retrospective cohort study of neonates with seizures revealed that 50% of neonates with stroke affected by focal seizures (7). In Lynch et al. study, all 6 term neonates affected with stroke had a focal clonic seizure that was similar to our results (6). Estan et al. in investigation of 12 newborns suffered from stroke found that 8 cases had focal clonic seizures and three had generalized clonic and the remaining neonates had subtle seizures (5). Electroencephalographic (EEG) results in neonatal stroke may be normal or show sharp and spike waves, focal slowing, or periodic lateralized epileptiform discharges (9). EEG findings in all affected cases of our study with stroke were abnormal but in 3 of 11 cases of Laugesaar study and in 20 from 48 neonates of Rafay et al. study were abnormal (7, 8). Evaluation of risk factors for neonatal stroke in our study revealed that umbilical venous catheterization has been increased the risk of stroke. Unfortunately, a few casecontrol studies have been carried out to identify risk factors of neonatal stroke and the etiology and prognosis of neonatal stroke have not been exactly described. Therefore, this is a large field for further evaluation (8, 21). Dajani et al. introduced 2 cases of neonatal stroke associated with portal vein thrombosis. They proposed that in the absence of any identifiable etiologies for the neonatal stroke, portal vein thrombosis should be considered and a Doppler ultrasonography of portal system was recommended because the authors believed that the source of thromboembolism commonly was undetermined. Instrumentalism, particularly umbilical venous vein catheterization is the most common cause of portal vein thrombosis (9). Although we did not carry out portal vein ultrasonography, our results are partly confirmed Dajani et al. conclusion about association of umbilical vein catheterization with increased risk of neonatal stroke. This association has been demonstrated in other studies and umbilical venous catheterization is a well-known risk factor that increased risk of thrombovascular events and neonatal stroke (10, 18, 22). Govaret et al. in a retrospective cohort study of 134 neonates with stroke, reported that embolism was the most common cause of stroke (in 25% of cases) and the source of embolism were twin-twin transfusion, exchange transfusion and umbilical venous catheterization with air embolism (10). In our study, congenital heart disease has not increased the risk of neonatal stroke. Although congenital heart diseases are a known risk factor for ischemic stroke in neonates, it is less commonly implicated in the etiology of neonatal stroke and usually has been seen in cases who tolerated cardiac surgery and silent infarction may occur. On the other hand, stroke with cardiogenic origin is the only type of stroke, which need to anticoagulant therapy (7, 18, 22). We had a number of limitations in our study. First, not all of the neonates were tested for specific prothrombotic factors such as Pr C, S, antitrombin III, anti cardiolipin antibodies, lupus anticoagulant and factor V leiden and we might have missed some risk factors of stroke. Second, in an ideal condition, an MRI would be done first followed by a magnetic resonance angiogram but we did nott request MRI as an initial step and MRI was carried out for patients only according to results of Doppler ultrasonography because MRI was not available in our hospital and we had to give sedation to the neonates and transferred them to another hospital that was far from our center that it was impossible for all neonates. Therefore, we used Doppler ultrasonography as an initial investigation. Third, the follow up of most of the neonates with stroke was not completed for further evaluation and investigation of outcomes. In conclusion, the prevalence of 3.4% for diagnosis of stroke in MRI reinforced the importance of neonatal cerebral infarction as an important etiology of neonatal seizures. In addition, the noninvasiveness and portability of Doppler ultrasonography can promote its use in diagnosis of stroke, especially in monitoring of the critically ill infants and in conditions that MRI is not easily accessible. Further studies with more sample size and assessment of other risk factors, outcome assessment and determination of prognosis in neonatal stroke are recommended.
